# Landmark-free statistical shape modelling reveals effects of age and sex on whole muscle morphology among the triceps surae

**DOI:** 10.1098/rsos.250198

**Published:** 2025-04-09

**Authors:** India Lindemann, Robert L. Cieri, Christofer J. Clemente, Taylor J. M. Dick

**Affiliations:** ^1^ School of Biomedical Sciences, University of Queensland, Brisbane, Queensland, Australia; ^2^ Department of Zoology, University of British Columbia, Vancouver, British Columbia, Canada; ^3^ University of the Sunshine Coast, Sippy Downs, Australia

**Keywords:** ageing, gastrocnemii, geometric morphometrics, magnetic resonance imaging (MRI), skeletal muscle

## Abstract

The shape of skeletal muscle has important influences on muscle function, yet studies of three-dimensional shape variation are rarely performed. Analysis of muscle shape variation using traditional tools is limited by lack of anatomical landmarks, but modern landmark-free methods provide new opportunities to study complex shapes. We used generalized Procrustes surface analysis to characterize shape variation among the triceps surae: medial gastrocnemius (MG), lateral gastrocnemius (LG) and soleus (SOL), digitized using magnetic resonance imaging from 21 younger (8 females, 13 males; 24.6 ± 4.3 years) and 15 older (6 females; 9 males; 70.4 ± 2.4 years) physically active participants. In both gastrocnemii, the first principal component (PC) of shape variance was related to muscle width and thickness. The second PC was related to variation in the MG’s insertion and variation in thickness along the LG long axis. In the SOL, the first PC was related to overall muscle thickness and length while the second PC captured variation in lateral margin thickness and curvature of the medial border. Muscle shape differed between young and older adults in MG and LG, while SOL shape differed between males and females. These findings demonstrate statistical shape modelling as a promising tool for disentangling multiple influences on skeletal muscle shape and provide important input for future biomechanical modelling investigations.

## 1. Introduction

Skeletal muscle morphology is closely linked to its function, with variations in shape and size reflecting differences in mechanical roles. For instance, despite similar volumes, the sartorius and lateral gastrocnemius (LG) differ markedly in their force production capabilities due to distinct architectural features: the sartorius has long fibres enabling large excursions, while the LG’s short, pennate fibres and larger physiological cross-sectional area (PCSA) optimize force generation [1]. Advances in imaging have revealed how muscle architecture changes across age groups [2], sexes [3] or with neuromuscular disease [4]. Ageing, for example, is associated with reduced muscle mass and function, shorter fascicles and lower pennation angles, though these changes vary between muscles and are influenced by activity levels [5,6]. Sex differences in muscle architecture also exist, with females generally exhibiting thinner triceps surae muscles with longer fibres and lower pennation angles than males [3]. Despite these insights and the intimate links between muscle shape and performance, we have a limited understanding regarding how three-dimensional muscle shape varies among healthy individuals.

Skeletal muscles are complex three-dimensional structures, yet most analyses of their shape have been limited to scalar metrics such as muscle length, width or volume. Geometric morphometrics, also known as statistical shape analysis, uses multivariate statistical analysis to analyse and compare the whole configuration of an object, rather than traditional morphometrics, which attempts to characterize complex three-dimensional shapes with a combination of scalar metrics [7]. Using geometric morphometrics, it is possible to (i) remove the effect of size variation so that shape changes can be studied directly and (ii) reduce the complexity of anatomical shape variation into a smaller set of principal components (PCs) which represent the main features of shape variation within or between populations. The development of landmark-free statistical shape modelling approaches, such as generalized Procrustes surface analysis (GPSA), enables this technique to be applied to objects without distinguishable features [8]. This approach may prove particularly important for the analysis of skeletal muscle, as it remains challenging to identify corresponding anatomical landmarks from magnetic resonance imaging (MRI) scans, which has previously limited our ability to perform statistical shape modelling using traditional landmark-based methods. Although landmark modelling may be possible in some skeletal muscles with prominent features that can be detected in MR images (e.g. aponeurosis of the tibialis anterior), it remains a challenge in most muscles. GPSA shape analysis has been used to examine diverse structures such as fossil crocodilian fragments [9], squamate brain architecture [10] and sea snail shell shape [11], but has not yet been applied to the shape analysis of skeletal muscle.

In humans, statistical shape modelling has been used to investigate variation in neuro-musculoskeletal structures such as bones [12], joints [13] and the brain [14], but there are few studies that have applied these modelling techniques to explore variation in skeletal muscle shape. In clinical populations, shape modelling has been used to identify differences in muscle morphology between cerebral palsy and typically developing individuals [15], to assess shoulder pathology based on three-dimensional muscle shape in the supraspinatus [16] and to investigate medial gastrocnemius fascicle shapes at different ankle positions [17]. Recently, Bolsterlee [18] combined shape modelling approaches with three-dimensional muscle architecture measurements from diffusion tensor imaging (DTI) to demonstrate the broad application of three-dimensional shape analysis ranging from athletes to clinical populations. Specifically, the authors incorporated a unique combination of traditional MRI measures, DTI and statistical shape modelling to explore the influence of strength-training interventions, passive contractions and cerebral palsy on three-dimensional muscle shape and morphology. Statistical shape modelling has not yet been used, however, to investigate three-dimensional morphological variation in multiple lower limb skeletal muscles. Application of this technique will provide insights into understanding how muscle shape varies both within healthy individuals and between populations with known differences in muscle architecture, such as ageing. Understanding the extent of this variation may provide important insights into altered muscle shape throughout the lifespan and could be translated to improve the diagnosis and treatment of muscle pathologies.

The aims of this study were to develop statistical shape models of the human triceps surae muscles (medial gastrocnemius (MG), lateral gastrocnemius (LG) and soleus (SOL)) to (i) characterize inter-individual variability in muscle shape within a population of young and older adults and (ii) determine the influence of age and participant characteristics (sex, height, body mass, muscle volume and physical activity) on individual muscle shape. To address these aims, we performed exploratory landmark-free geometric morphometric analyses on muscle surfaces segmented from MRI scans in 36 young and older female and male participants. We hypothesized that there would be substantial variability in three-dimensional muscle shape between individuals and that whole muscle morphology would vary between muscles among the triceps surae. We also expected that muscle shape would differ between young and older adults, distinguishable via one or more PCs of the shape models.

## 2. Methods

**Figure 1.**
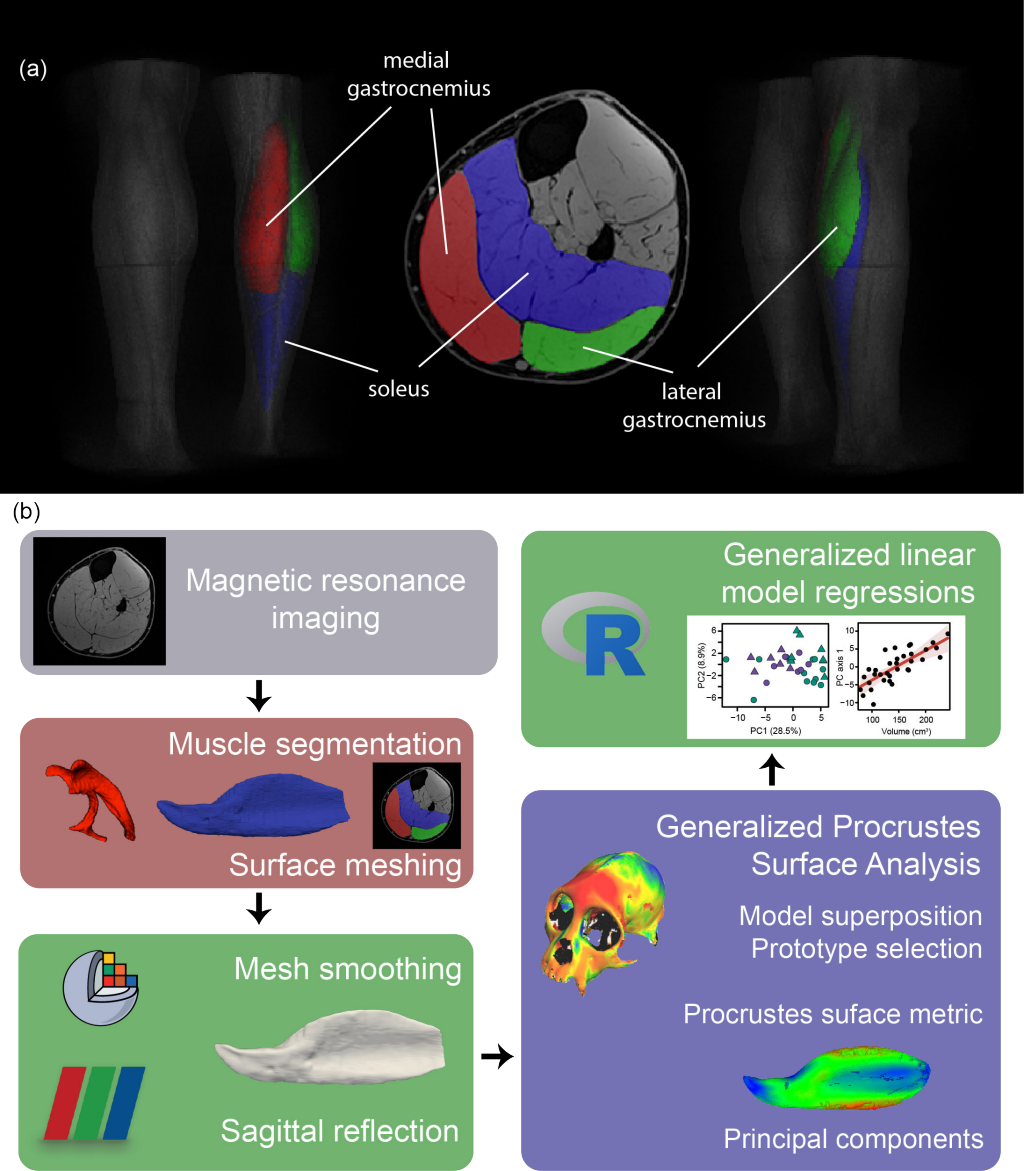
Overview of triceps surae anatomy and open-source methodological pipeline. (a) Segmentation of the right triceps surae musculature shown as a volume-rendered MRI scan (T1 VIBE with water excitation) from posterior-medial (left) posterior-lateral (right) views and as a transverse plane (centre), including medial gastrocnemius (red), lateral gastrocnemius (green) and soleus (blue). (b) Schematic of methods employed in this study showing major steps and software packages. Muscle segmentation was performed using both semi-automated and manual segmentation software. ITK-SNAP was used to create the surface meshes of the segmentations, which were smoothed in 3D Slicer at *σ* = 2 mm of Gaussian smoothing and with a surface smoothing factor of 0.5 applied when the surfaces were visualized in three-dimensional space. Left-leg muscle surfaces were reflected about the sagittal plane using Paraview. Pomidor *et al*.’s GPSA program was used for the statistical shape analysis [8]. The prototype muscle was selected to be the most ‘average’ muscle shape (see §2). Principal component analysis was performed on the interspecimen Procrustes surface metric distances and used for subsequent statistical analysis in R using the lm and step functions. We created general linear models for the first two principal components of each muscle and variables of interest and then performed backwards stepwise regression to determine the final models used for analysis.

A total of 36 participants including 21 younger (8 females, 13 males; 24.6 ± 4.3 years) and 15 older (6 females; 9 males; 70.4 ± 2.4 years) physically active adults were included in this study (table 1). Participant exclusion criteria included recent (6 months) lower leg injury as well as contra-indication to MRI (e.g. no pacemaker or specific metal implants), which was under the discretion of the radiographer. All participants provided informed consent, and ethics approval was obtained from the University of Queensland’s Human Research Ethics Committee (approval no. 2013001448). Participants were asked to self-report physical activity using the Active Australia Self-Report Physical Activity Measure [19] and those guidelines were used to determine the metabolic equivalent number of minutes (METmins) of activity per week. Ankle plantarflexion strength was measured on an isometric dynamometer (as per fig. 1*a* in [20]. Participants lay prone while their dominant limb (knee fully extended and ankle at 90°) was rigidly secured in a custom frame comprising a steel foot plate and a torque sensor (Model TRE-50K, Dacell Co., Ltd, Korea). Following a period of familiarization, participants performed three isometric plantarflexion maximal voluntary contractions (MVCs) with 120 s rest between each. Maximum ankle plantarflexion torque, a measure of strength, was taken as the maximum torque generated during the three contractions. Figure 1 outlines the workflow including (i) MRI acquisition and processing and (ii) generation and analysis of statistical shape models, which is described in detail below. The software utilized within our analysis pipeline is open-source.

**Table 1.** Participant characteristics.

	age (yrs)	height (cm)	body mass (kg)	BMI (kg m^−2^)	maximum ankle plantarflexion torque (Nm)	physical activity (METmins/wk)
young (*n* = 21)	24.6 ± 4.3	173.0 ± 8.9	71.6 ± 14.8	23.8 ± 3.4	126.5 ± 35.3	4051.7 ± 2791.8
older (*n* = 15)	70.4 ± 2.4	169.3 ± 10.1	72.7 ± 17.1	25.2 ± 4.4	88.1 ± 38.6	2394.9 ± 1200.7
*p*‐value	*p* < 0.001	*p* = 0.116	*p* = 0.868	*p* = 0.356	*p* < 0.001	*p* < 0.001

Values are expressed as mean ± s.d. BMI: body mass index. Presented previously in [6]. To assess differences between groups, *t*‐test or Mann–Whitney test was performed.

### 2.1. Magnetic resonance imaging protocol

Participants lay supine in a 3T MRI scanner (Magnetom Prisma, Siemens, Germany). Their dominant leg (self-reported) was secured in a custom foot plate with the ankle at 90°, and a foam wedge was positioned under the knee to minimize deformation of the calf muscles due to an interaction with the surface of the MRI bed. An 18-channel body matrix receive-only coil, in conjunction with the built-in spine coil, were used to acquire the images. Two hundred and sixty transverse anatomical slices were obtained, covering a region proximal to the knee joint and distal to the ankle joint to ensure visualization of the whole triceps surae muscle group. The T1-weighted MRI settings for our protocol have been previously described [6] and are briefly presented here. The scan parameters were as follows: Siemens T1 VIBE, field of view (FOV) = 262 × 350 mm, acquisition matrix 336 × 448 (reconstructed matrix = 672 × 896), slice thickness = 2 mm, 260 slices (no gap between slices), repetition time (TR)/echo time (TE) = 11.7/5.29 ms, flip angle = 10°, number of signal averages (NSA) = 1, scan time = 7 min 42 s (2 sequences to cover entire length of shank at 2 min, 51 s each). The MRI scans were analysed using a combination of semi-automated (Sashimi v. 1.1 [21]) and manual segmentation software (ITK-SNAP v. 3.8.0, NIH, USA). Further analysis details are reported in [6].

Surfaces meshes of the segmented regions of interest were created in ITK-SNAP, and refined via Gaussian smoothing (2 mm) and a surface smoothing factor of 0.5 when visualized in three dimensions (electronic supplementary material, figure S1) (3D Slicer v. 4.11.20210226 [22]). This smoothing was chosen as it appeared to strike the balance between smoothing segmentation artefacts and preserving muscle shape information compared with other smoothing strengths (1 and 3 mm smoothing were also explored) [23]. Two SOL meshes were smoothed at 3 mm due to surface artefacts introduced with *σ* = 2 mm smoothing (electronic supplementary material, figure S1). The LG for one participant was removed from analysis due to segmenting artefacts that were unable to be resolved while preserving muscle shape information. Surface meshes for scans that were acquired on the left leg (*n* = 7) were reflected about the sagittal plane (Paraview v. 2021.07 [24]); to ensure that all muscles were in the same anatomical frame of reference for shape analysis.

### 2.2. Statistical shape analysis

We used GPSA on the smoothed surfaces to obtain shape models for each muscle [8]. GPSA is an open-source, landmark-free statistical shape analysis software. Briefly, GPSA generates shape models by selecting an initial reference surface, defined as the prototype, superimposing all other surfaces to the reference using an iterative closest point algorithm, and then updating the reference shape each iteration to create an average surface. The similarity of any two surfaces is then computed by a distance metric called the Procrustes surface metric, which is analogous to the familiar Procrustes distance from landmark-based geometric methods [8]. This Procrustes surface metric is similar to a root mean square distance metric (Procrustes distance) but weights the points on each surface to ensure symmetry, as different surfaces can have different numbers of points. Specifically, the distance between 297 852 landmark points was used for the MG, 211 707 for the LG and 373 806 points for the SOL. Following this, principal component analysis is performed by the GPSA program on the interspecimen Procrustes surface metric using Gower’s principal coordinates analysis (PCOORD) method [25] to determine the main axes of shape variation. The PCA ordination axes coordinates for each muscle for each individual were computed as variation from the average muscle shape model and used for subsequent statistical analysis.

To create the average muscle shape surface, we first selected a prototype surface which was then reformed to fit the final distribution of points in the set of superimposed surfaces. Pomidor *et al*. [8] highlight that this prototype surface should be the most complete, most representative and least morphometrically atypical individual. To determine which surface mesh should be used as the prototype file, the GPSA pipeline was repeated for all 36 potential surfaces and a sensitivity analysis was conducted to determine the relative effect of the prototype surface by examining the per cent variance explained by each of the first four PCs. Results of the sensitivity analysis demonstrated that the prototype file had an influence on the PC scores (electronic supplementary table S1). Therefore, the prototype surface for the final shape analysis in the MG, LG and SOL was determined based on the individual muscle which had the lowest interspecimen Procrustes surface metric distances compared with all other surfaces (i.e. the shape most similar to all other shapes—the most ‘average’ shape). However, for the MG the individual with the lowest interspecimen values produced heat maps displaying non-physiological anomalies in the shape of the muscle, so based on Pomidor *et al*. [8], the participant with the second lowest interspecimen value was selected as the prototype file.

### 2.3. Comparison of statistical shape models

To determine the relationship between age, sex, height, mass, muscle volume and METmin of physical activity on three-dimensional muscle shape, general linear models (GLMs) were developed for each muscle and backward stepwise regression was performed for each of the first two PCs (R v. 4.1.2) using the ‘lm’ and ‘step’ functions. First, normality assumptions were checked by visual inspection of the quantile–quantile plot. Model parameters included log(mass), age, sex, muscle volume, METmin, height and the interaction of age and sex. After backwards stepwise regression was performed, the models for each muscle for PC1 and PC2 only included terms that had a significant effect. Electronic supplementary material, table S2, provides the final GLMs used in analysis. Significance was set as *p* < 0.05.

## 3. Results

**Figure 2.**
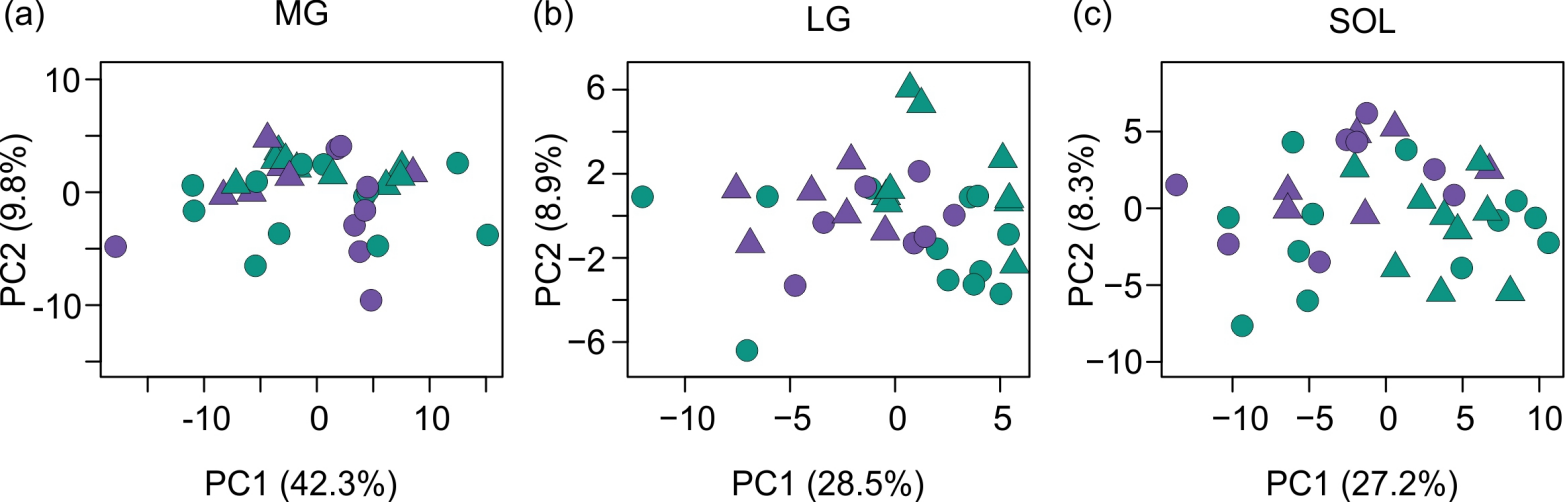
PC1 versus PC2 weights for the medial gastrocnemius, lateral gastrocnemius and soleus of all participants. (a) In the medial gastrocnemius (MG), principal component 1 (PC1) explained 42.3% of shape variance and principal component 2 (PC2) explained 8.9% of shape variance. (b) For the lateral gastrocnemius (LG), PC1 explained 28.5% of shape variance and PC2 explained 8.9% of shape variance. (c) For the soleus (SOL), PC1 explained 27.2% of shape variance and PC2 explained 8.3% of shape variance. Each data point represents an individual, with young and older adults denoted as circles and triangles, respectively. Sex is indicated by colour with females shown in purple and males in green.

**Figure 3.**
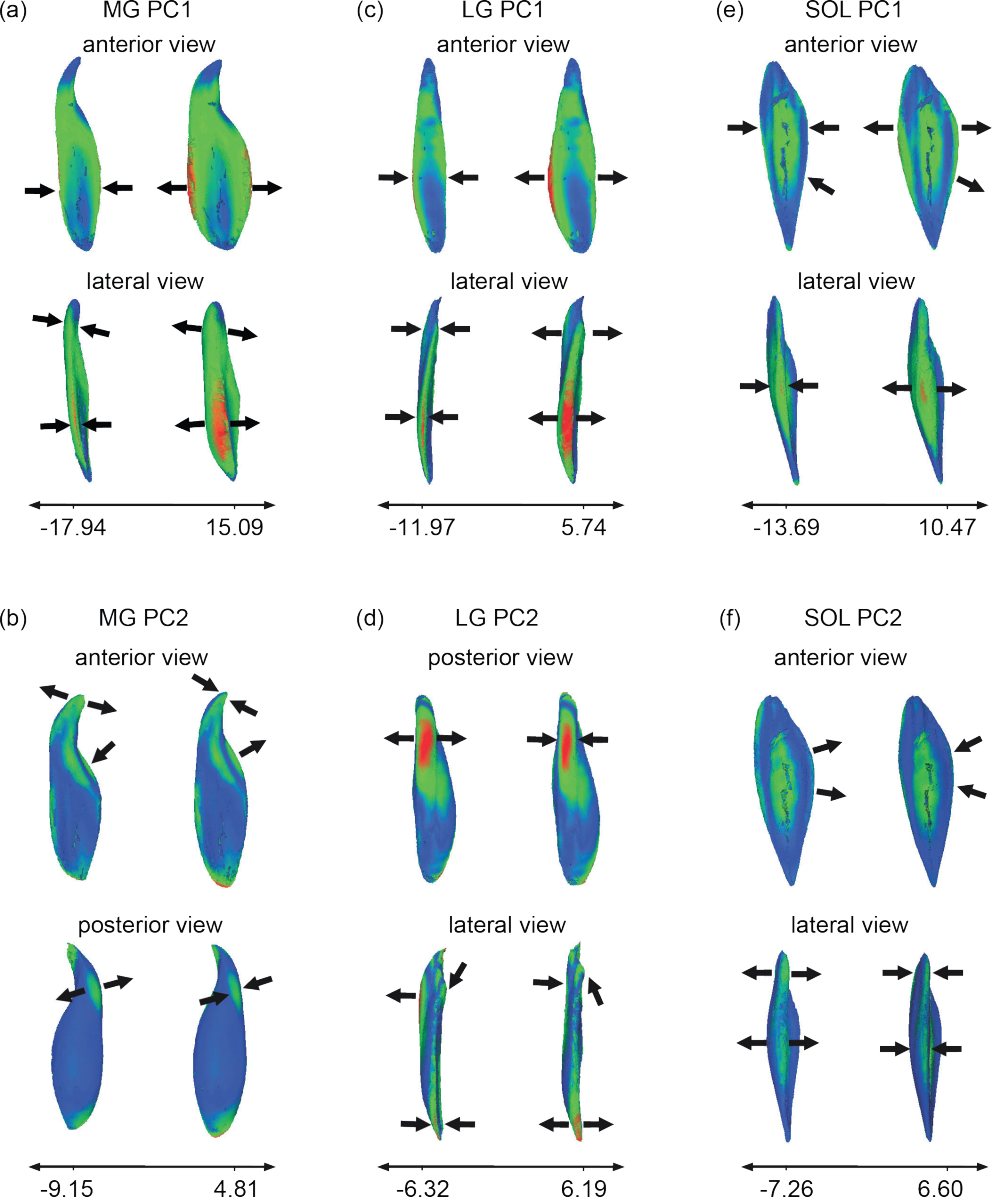
The differences in shape of the medial gastrocnemius (MG), lateral gastrocnemius (LG) and soleus (SOL) associated with the minimum and maximum of the first two principal components (PC1 and PC2). These models represent theoretical projections of shape based on maximum and minimum PC scores and are generated by Pomidor *et al*.’s open source GPSA program [8]. Anterior and lateral views of PC1 in the MG, LG and SOL are shown (a,c,e). Anterior and posterior views of PC2 are shown for the MG (b), while posterior and lateral views are shown for the LG (d) and anterior and lateral views are shown for the SOL (f). Colour represents the level of variation in the shape, from low (blue) to high (red). Arrows depict notable areas of shape differences, with arrow direction indicating the direction of shape difference as you move along the PC axis.

The first PCs explained a great deal of variation in all three muscles of the triceps surae (figure 2). The percentage of variance in shape explained by PC2 was lower in the soleus than the MG and LG. The principal shape differences are visualized in figure 3 and electronic supplementary material, video S1.

### 3.1. Medial gastrocnemius

The first 10 PCs accounted for 78.5% of the overall variance in MG shape, while the first two PCs accounted for 52.1% of the overall variance in shape (electronic supplementary material, figure S2). PC1 is related to the combination of variation in (i) muscle width (in the medial-lateral direction) in the distal half of the muscle and (ii) muscle thickness (in the anterior-posterior direction). This means that larger PC1 values represented a wider and thicker muscle (figure 3a). In the MG, PC2 captured variation in the shape of the proximal tendinous junction, with lower PC2 scores representing more axial torsion in the region where the MG crosses the knee joint to insert on the posterior surface of the medial femoral condyle (figure 3b).

### 3.2. Lateral gastrocnemius

For the LG, 72.5% of the cumulative variance was accounted for in the first 10 PCs, while the first two PCs accounted for 37.3% of the overall variance in shape (electronic supplementary material, figure S2). Consistent with the MG, PC1 for the LG represented variation in muscle width and thickness, with the highest variability on the anterolateral distal half of the muscle (figure 3c). Lower PC1 scores represented an LG with a more constant width along its proximal to distal length, whereas higher PC1 scores were linked to a muscle with a more variable thickness and width. Lower PC2 scores were associated with a thicker LG (figure 3d). Variation in PC2 for LG was greatest at the proximal posterior surface of the muscle and at the distal lateral margin.

### 3.3. Soleus

The first 10 PCs accounted for 69.2% of the overall variance in SOL muscle shape, while the first two PCs accounted for 35.5% of the overall variance in shape (electronic supplementary material, figure S2). Lower PC1 values were associated with a thinner and more elongated SOL (figure 3e). Lower PC2 scores for SOL were related to a thicker lateral margin and a wider muscle curving around the medial border (figure 3f).

### 3.4. Variability in shape with age and participant characteristics

**Figure 4.**
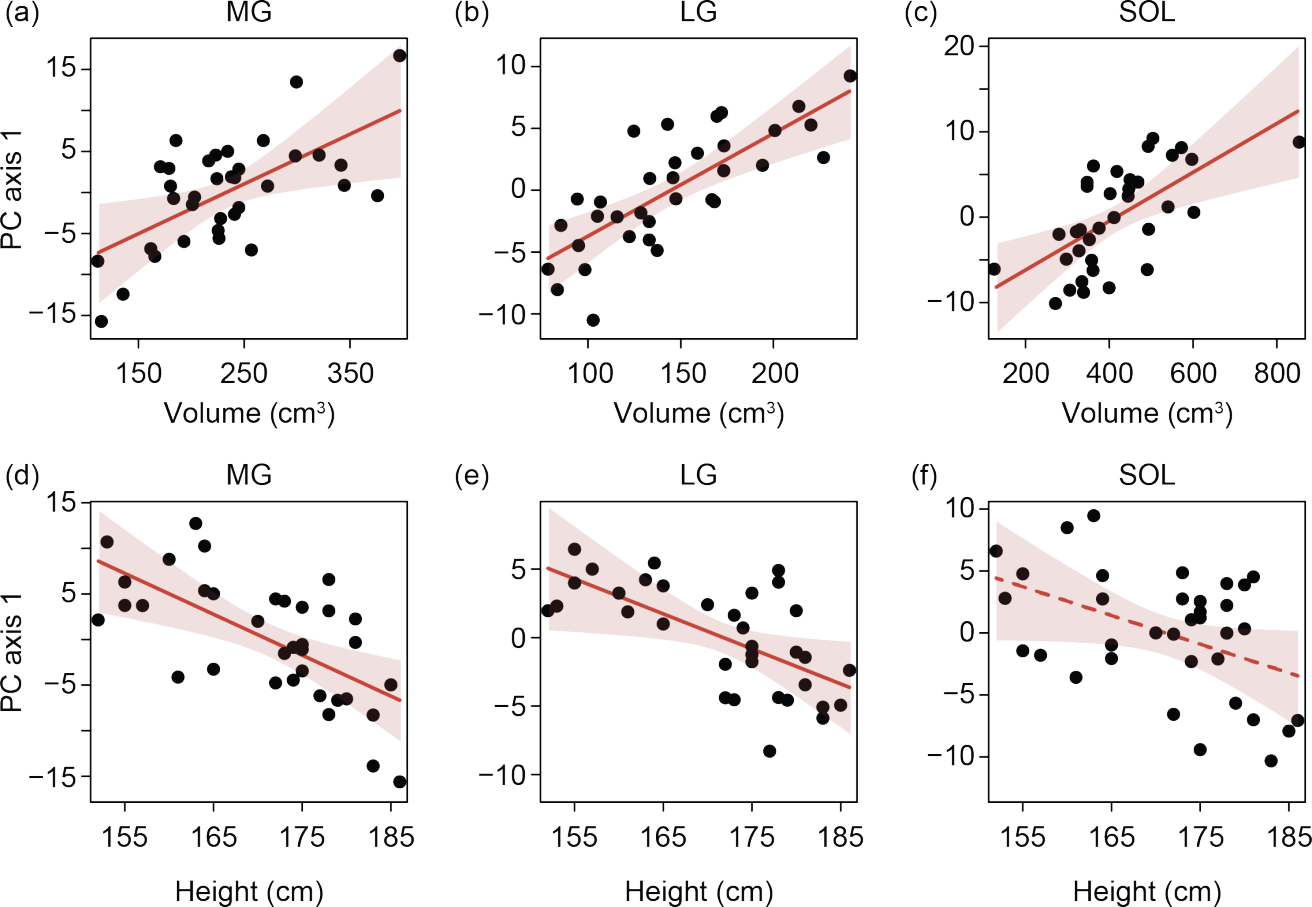
Shape differences associated with muscle volume and participant height across the triceps surae. Data are presented as residual effect plots. Muscle volume is positively associated with PC1 scores across the triceps surae ((a) medial gastrocnemius (MG) *r*^2^ = 0.18, *p* = 0.014; (b) lateral gastrocnemius (LG) *r*^2^ = 0.42, *p* < 0.001; (c) soleus (SOL) *r*^2^ = 0.27, *p* = 0.002), while participant height is negatively associated with PC1 scores for the MG and LG ((d) MG *r*^2^ = 0.26, *p* = 0.002; (e) LG *r*^2^ = 0.17, *p* = 0.025), but not SOL ((f) *r*^2^ = 0.11, *p* = 0.055). For each relationship (a–f), partial regressions (red line) are shown from a general linear model to the partial residual data (black circles). Solid red lines (a–e) indicate statistical significance (*p* < 0.05), dashed red lines (f) indicate no statistical significance (*p* > 0.05). The red shaded area represents the 95% confidence intervals. The function ‘partial_r2’ from the sensemakr package in R was used to calculate partial *r*^2^ values.

**Figure 5.**
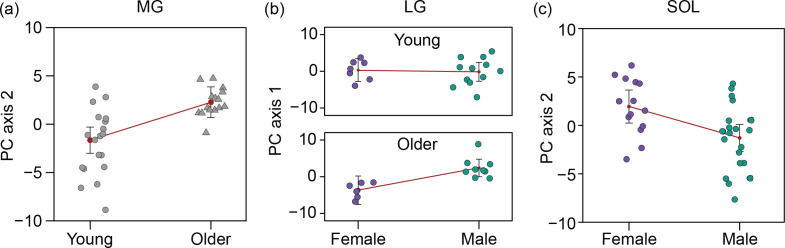
Shape differences associated with age and sex across the triceps surae. Data are presented as residual effect plots. Medial gastrocnemius (MG) PC2 scores were significantly higher in older adults ((a) *p* < 0.001). Lateral gastrocnemius (LG) PC1 was significantly associated with age ((b) *p* = 0.037) and the interaction between age and sex ((b) *p* = 0.008). Soleus (SOL) PC2 was higher in females compared with males ((c) *p* = 0.005). For a visualization of the relationships of both PC axes with age and sex across all three muscles including non-significant associations, please see electronic supplementary material, figure S4.

We found a significant positive effect of muscle volume on PC1 for all three muscles (MG: *p* = 0.014; LG: *p* < 0.001; SOL: *p* = 0.002) (figure 4a–c). Height was negatively associated with PC1 in the MG (*p* = 0.002) and LG (*p* = 0.025), but not the SOL (*p* = 0.055) (figure 4d–f). This means that muscles with greater volumes were thicker and wider, and taller individuals possess more elongated muscles. There were no significant effects of muscle volume and participant height on PC2 scores (electronic supplementary material, figure S3). Age, sex and their interaction had a significant influence on variation in shape for some, but not all, muscles. Older adults had higher MG PC2 values than younger adults (young: −1.4 ± 3.7; older: 1.8 ± 1.4; *p* < 0.001) (figure 5a). This translates to an MG in older adults that was characterized by less axial torsion in its proximal region. LG PC1 values were significantly associated with both age (*p* = 0.037) and the interaction between age and sex (*p* = 0.008), meaning that differences in LG PC1 between young and older groups, which corresponds to muscle thickness and width, depended on sex (figure 5b). Females had higher SOL PC2 values compared with males (female: 2.0 ± 2.9; male: −1.3 ± 3.3; *p* = 0.005) (figure 5c) meaning that the SOL in males was thicker and had a more curved lateral margin.

### 3.5. Sensitivity to prototype surface

The results of the GPSA were dependent on which participant was selected as the prototype muscle shape (the ‘mean’ surface), and the sensitivity to this prototype selection depended on the PC and muscle examined (electronic supplementary material, table S1). The first PC was most sensitive in SOL, where the amount of variation in shape explained by PC1 varied from 16% to 34% depending on the participant used as the prototype. PC2 scores were more sensitive in the MG than the LG and SOL, with the difference between the maximum and minimum per cent variation explained being 10 times larger than that of the LG and SOL (15% versus 1.9% and 1.7% for MG versus LG and SOL, respectively).

## 4. Discussion

In this study we developed statistical shape models of the human triceps surae muscles to assess how the three-dimensional shape characteristics of these muscles varied in both young and older adults. Landmark-free geometric morphometric analysis provides a promising approach to characterize variations in skeletal muscle shape from imaging data as it does not rely on the identification of corresponding landmarks which is often challenging or not possible in skeletal muscle. We were able to characterize complex variation in three-dimensional muscle shape, with the first two PCs accounting for 54%, 37% and 35%, of the overall variance of the shape of the MG, LG and SOL, respectively, and the cumulative variance from the first 10 PCs ranging from 69% to 78% across muscles. We found that three-dimensional muscle shape differed between young and older adults in the MG and LG, and that females and males possessed differences in SOL shape.

### 4.1. General patterns of shape variation

The variance of shape characteristics was similar for MG and LG, and differed only slightly in the SOL. The primary sources of variation corresponded to muscle thickness and width, and to a lesser extent muscle length. This has direct implications on muscle function, as an increase in width and thickness would lead to an increase in cross-sectional area, which is linked to a muscle’s force-generating capacity [26]. Visual inspection of the shape models (figure 3; electronic supplementary material, video S1) suggest less regular shape variation in the SOL, which is reflected by the fact that the first two PCs explain less of the total variance in the SOL compared with the MG and (to a lesser extent) the LG. Previous investigations of skeletal muscle architecture have shown greater variability in LG and SOL than MG for properties including muscle mass and PCSA [1] as well as muscle volume [6], which is, in part, consistent with our findings.

The variation of the MG along the second PC axis appears to physically represent alterations in axial torsion in the proximal region of the muscle. One study has explored the role that muscle shape plays in athletic performance via comparing hamstring shape in rugby and sprinting athletes [27]. They demonstrated differences in axial torsion between athlete groups and suggest that greater amounts of torsion may enable rapid power output and nonlinear movements—although this is yet to be experimentally confirmed. Future studies integrating MRI to determine muscle shape variation together with biomechanical measures to characterize locomotor performance may provide further insight. Variations in muscle shape may be related to the regional distribution of activation across a muscle. In both the MG and LG, there is a large degree of inter-individual variability in the regional distribution of EMG amplitude during knee flexion compared with ankle plantar flexion at matched global activation levels [28]. Combining information regarding muscle shape from imaging techniques and neuromuscular activation across different muscle regions from high-density surface electromyography [29] is an exciting area for further exploration—with implications for performance, injury risk, or pathology.

### 4.2. Influence of ageing on muscle shape

Here, we find age-related differences in muscle shape within the medial and lateral gastrocnemius, but no evidence for age-related differences in the soleus. There were no significant differences in shape with physical activity level. The primary sources of shape variation between young and older adults were axial torsion in the proximal region of the MG and muscle girth (thickness and width) for the LG. Although sharing a common function in ankle plantarflexion, the bi-articular MG and LG and uni-articular SOL differ in fibre type [30], architecture [1] and excitation [31]. Previous studies have demonstrated non-uniform age-related differences across the triceps surae. Morse *et al*. [2] showed that PCSA, estimated from muscle volume and fascicle length, was 14% and 19.5% smaller in the MG and LG of older compared with young males, respectively—however SOL PCSA was similar between groups. Pinel *et al*. [6] demonstrated that in physically active older adults, triceps surae muscle volumes were maintained or even slightly greater—albeit with a greater amount of intramuscular fat compared with young adults. It may be that SOL shape and architecture are relatively conserved through the ageing process in healthy, active older adults. A recent review by Naruse *et al*. determined that the gastrocnemii experience roughly three times the amount of atrophy as the soleus with ageing. The reasons for this are complex but probably due to a combination of fibre type differences between muscles and activity levels [32]. The cross-sectional area of type II fibres declines with age, which does not seem to be the case with type I fibres [33]. The soleus, containing a higher proportion of type I fibres [34], would therefore be less likely to experience shape changes owing to alterations in fibre size. Additionally, the soleus is considered a postural muscle, thus is more often active in everyday tasks such as balance and walking compared with the gastrocnemii [35]. Thus, greater shape changes in the gastrocnemii with ageing may be expected considering the lower activity levels of older adults, both in general and within our cohort (table 1).

### 4.3. Muscle shape in motion

Static muscle shape is probably linked to the way a muscle deforms during a contraction, which has important implications for muscle performance [36,37]. Ageing influences the dynamic shape changes that skeletal muscles undergo during contraction. For example, older humans have been shown to experience reduced muscle belly gearing, defined as the ratio of muscle belly velocity to muscle fascicle contraction velocity, which has a negative effect on overall mechanical output [20,38]. The differences in muscle shape between older and younger adults identified in this study may have important implications for understanding muscle performance with advanced age. Future efforts that combine shape analysis with muscle architecture and mechanical properties within three-dimensional biomechanical models are probably needed to explore these ideas. This is, in part, because ageing is complex and the multiple factors that influence impaired neuromuscular function are challenging to either isolate *in vivo* or to characterize simultaneously. Statistical shape models of skeletal muscle, such as those presented here, may be an effective way to personalize muscle models of the lower limb to be used within musculoskeletal models of human ageing [39,40].

### 4.4. Variation between females and males

Soleus muscle shape varied with sex, with males possessing thicker and wider SOL muscles compared with females. There was no evidence for an effect of sex on MG or LG muscle shape variation; however, a significant age*sex interaction demonstrated that LG shape was similar in young males and females, but differed between older males and females (figure 5b). Specifically, the LG in older males showed a larger girth (thickness and width) compared with older females. These sex-differences are consistent with studies that have used B-mode ultrasound to quantify muscle thickness in the triceps surae. Chow *et al*. [3] measured muscle thickness at multiple regions in the MG, LG and SOL and found that the greatest percentage difference of muscle thickness between males and females was in the proximal part of the posterior soleus. The lack of evidence for a sex difference in MG shape is also in agreement with a previous study in 121 men and 190 women that showed sex-related differences in muscle thickness for vastus lateralis and triceps brachii, but not MG [41].

Here we have investigated skeletal muscle shape, which is just one characteristic of muscle morphology. Future research that combines information from different imaging modalities, for example muscle shape and size from MRI and muscle architecture from DTI [18], will enable a more comprehensive understanding of how muscle function is influenced by ageing (young versus older); disease (healthy versus pathological); or evolution (species comparison). Importantly, and owing to their iso-volumetric nature, muscles change shape when they contract—bulging in three dimensions. Numerous studies have quantified the two-dimensional shape changes (i.e. length, thickness) that are associated with different contraction intensities [37,38], and may be influenced by ageing [20,42], stroke [43], or training interventions [44]. Quantifying *in vivo* three-dimensional muscle deformations remains challenging because scanning times often exceed the duration over which an individual can maintain a constant force or torque level, and movement artefacts owing to force fluctuations may disrupt the MR images. Despite these challenges, one study created three-dimensional surface models of the MG from MRI scans in a small number of individuals during plantarflexion contractions at intensities of 10% and 20% MVC maintained for 2.5 min, and also while the muscle was relaxed [45]. This approach provides encouraging avenues for characterizing three-dimensional deformations during contractions in the near future.

### 4.5. Limitations

There are some limitations of our study that should be acknowledged. First, GPSA relies on the selection of a prototype surface and our shape analysis results were sensitive to which participant was used as this ‘average’ muscle shape. Although this is perhaps not surprising given the significant variation in muscle shape observed across our cohort, our sensitivity analysis was purposefully conducted to quantify this effect. Future studies should consider the chosen surface, and whether a specific population (i.e. older adults) should be compared with an average young muscle rather than one specific individual. Second, our MRI protocols were conducted with the muscles in a rested state; however, information regarding muscle shape during contraction may provide additional information linking muscle shape and function. Future studies could endeavour to create three-dimensional surface models from MRI of muscle in a contracted state [45]. Third, our sample size was 36 participants consisting of 21 younger and 15 older adults which enabled us to capture the key features of three-dimensional muscle shape in young and older adults; however, a larger sample size across a greater ageing phenotype from frail to fit may provide more information that can be generalized across the ageing spectrum.

### 4.6. Conclusions

In this study, we developed statistical shape models of the triceps surae muscles in young and older adults. Our results demonstrate substantial variation in muscle shape related to ageing and sex in some, but not all, muscles of the triceps surae. Landmark-free statistical shape modelling enables us to more clearly discriminate whole muscle morphology in ageing or pathological populations at a resolution previously not possible. Knowledge of the inter-individual or population-level variations in skeletal muscle shape can be incorporated within future biomechanical models to explore detailed relationships between skeletal muscle form and function. Future work should investigate how these static shape differences may be linked to age-related differences in muscle deformations and fascicle behaviour during dynamic tasks that are most relevant to everyday locomotor function.

## Data Availability

The data that support the findings of this study are openly available in [46]. Supplementary material is available online [47].
